# Analysis of the Microbiome of Rainbow Trout (Oncorhynchus mykiss) Exposed to the Pathogen Flavobacterium psychrophilum 10094

**DOI:** 10.1128/MRA.01562-19

**Published:** 2020-03-19

**Authors:** Natalia Valdés, Alex Gonzalez, Veronica Garcia, Mario Tello

**Affiliations:** a Bacterial Metagenomics Laboratory, Faculty of Chemistry and Biology, Universidad de Santiago de Chile, Santiago, Chile; b Environmental and Extremophiles Microbiology Laboratory, Department of Biological Sciences and Biodiversity, Universidad de Los Lagos, Osorno, Chile; c Department of Food Science and Technology, Faculty of Technology, Universidad de Santiago de Chile, Santiago, Chile; Loyola University Chicago

## Abstract

Rainbow trout that were resistant or susceptible to Flavobacterium psychrophilum infection were compared with respect to their microbial composition by using 16S rRNA V3-V4 sequencing. The differences occurred in gills, where resistant fish displayed a greater abundance of the phylum *Proteobacteria* and a smaller proportion of *Firmicutes* relative to those of susceptible fish.

## ANNOUNCEMENT

The microbial composition of fish and other organisms plays an important role in the development of diseases caused by pathogens (1, 2), making the host more resistant or susceptible to certain diseases (3, 4). Flavobacterium psychrophilum is the etiologic agent of cold-water disease and rainbow trout fry syndrome, with mortality rates of over 50% (3). Currently, survival is attributed to natural diversity in the immune capacity of each infected individual (3) and the pathogen strain involved (5). However, the exact reason for this phenomenon is not well understood. The microbiome is a first barrier against the establishment of pathogenic bacteria within tissues and shows variation that depends on the history of each specimen (e.g., genetic, environmental, and feeding history) (6–9), but its protective role regarding F. psychrophilum infections has not been investigated. Thus, we hypothesized that resistant fish possess distinctive microbiome features that support their resistance to the infection/disease produced by this pathogen, as well as their eventual survival.

To obtain samples from Oncorhynchus mykiss fish that were resistant or susceptible to Flavobacterium psychrophilum, we applied an experimental challenge model using healthy fish from a fish farm located in Río Blanco, Chile. The challenge was performed with 60 specimens, each of ∼30 g, distributed in four 27-liter aquariums (15 fish per aquarium) at 14°C. The fish from two aquariums (30 fish in total) were injected intraperitoneally with live F. psychrophilum 10094 (10^8^ CFU/fish) to induce a pathogen challenge, while the fish from the other two aquariums (30 fish) were used as controls for the challenge. Next, the fish were kept in the facilities of the Center for Aquaculture Biotechnology of Universidad de Santiago de Chile for 20 days in 27-liter aquariums at 12°C and a density of 16 g/liter.

The fish were sacrificed and, immediately afterward, samples of gills, intestines (stool free), and stools (intestinal contents) were collected under sterile conditions from fish not injected with the pathogen, fish that developed the disease, and fish that showed no symptoms of the disease. Organs and stools were extracted in full, and 30 mg was used for DNA extraction. Five fish per condition were sampled.

After sample extraction, total DNA was extracted, using the genomic DNA purification kit (Promega), from each of the extracted organs and stool. DNA samples were pooled according to condition and organ, yielding 9 groups in total. DNA was frozen at –20°C until the samples were sent to the sequencing service.

Macrogen (South Korea) performed next-generation sequencing of the V3-V4 region of the 16S rRNA gene with the primers 341F (5′-CCT ACG GGN GGC WGC AG-3′) and 785R (5′-GAC TAC HVG GGT ATC TAA TCC-3′) (10) using the Illumina MiSeq platform. After sequencing, reads of approximately 250 bp were obtained; the average number of paired-end reads obtained was about 490,000. The numbers of paired-end reads per sample varied between 626,000 in the intestines of resistant fish and 379,000 in the stools of susceptible fish. Then, the QIIME v2 (11) next-generation microbiome analysis platform was utilized. Specifically, the DADA2 v0.99.8 plugin (12) for quality control was used for denoising (parameters: p-trunc-len-f 280 and p-trunc-len-r 240) and trimming, in which low-quality sequences were eliminated, decreasing the numbers of paired-end reads to 316,000 in the intestines of resistant fish and 297,000 in the intestines of susceptible fish; finally, the SILVA v132 database (13, 14) was used for taxonomic assignment, with similarity of 99% as the threshold.

**FIG 1:**
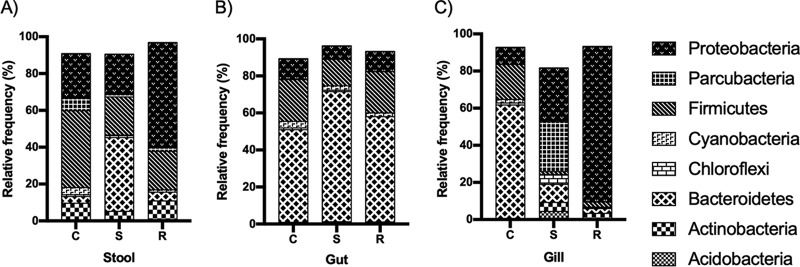
Relative frequencies of bacterial phyla in fish samples, as detected by metagenomic analysis. The samples analyzed correspond to stools (A), gut (stool free) (B), and gills (C) of fish resistant to Flavobacterium psychrophilum (R), fish susceptible to infection (S), and control fish (C). Only phyla with an abundance of ≥5% are shown.

Figure 1 summarizes the most abundant phyla (>5%) that were detected. *Bacteroidetes* showed an abundance of 61.46% in control gills, while the gills of resistant and susceptible fish showed abundances of 2.57% and 9.74%, respectively. In intestines, this phylum showed an abundance of 50.02% in control fish; in susceptible and resistant fish, its abundances were 70.35% and 56.99%, respectively. In stools from control fish, *Bacteroidetes* had an abundance of 3.68%, which changed to 4.67% in resistant fish and 40% in susceptible fish. The phylum *Firmicutes*, in contrast, decreased after exposure of the fish to F. psychrophilum 10094, regardless of the tissue analyzed. Finally, the phylum *Proteobacteria* increased its abundance in gills from 9.31% in control fish to 83.71% in resistant fish.

The microbiotas of fish that are resistant versus those that are susceptible to infections with Flavobacterium psychrophilum 10094 differ mainly in the abundance of the bacterial phylum *Proteobacteria*, which is found in greater abundance in the microbiota of resistant fish, compared to the microbiota of those that are susceptible to the pathogen.

### Data availability.

This metagenomic project has been deposited in the SRA under BioProject accession number PRJNA557254.
